# LOVOT: LOVE + ROBOT

**DOI:** 10.1002/alz.084181

**Published:** 2025-01-09

**Authors:** Zheng Yi Low

**Affiliations:** ^1^ SingHealth Community Hospitals, Singapore Singapore

## Abstract

**Background:**

Social robots have been used in other countries for improvement of quality of life for persons with dementia.

**Method:**

LOVOT was introduced as an adjunct to regular therapy sessions (either Physiotherapy or Occupational Therapy) and as an interactive companion during the patient’s inpatient stay. The project was carried out over a span of 6 weeks (weekdays) for a maximum of 10‐15 mins on an ad‐hoc basis.

**Result:**

A total of 19 participants were recruited over this 6 weeks. These included 11 patients (age between 49 to 81 year old, 6 with formal diagnosis of dementia and 5 without one). The other 8 participants were staff members (age 29 to 57 year old, ranging from the medical doctors to nurses to allied health staff). We administered one common questionnaire on both groups of participants, this questionnaire is “Sociability of robot”. There is 3 components to this questionnaire and they are as follows:

1. Perceived sociability. This component received 78% positive feedback from the participants

2. Social influence, which received 74% positive feedback

3. Social presence, which had 70% positive feedback. However it is worth noting that 54% of the participants felt that LOVOT was not a real pet during the interaction.

We also administered a second questionnaire: Older People’s Quality of Life (QoL) for the group of patient participants. There is a total of 13 statements. Most statements received positive agreement and 2 statements that had higher disagreement (36%) were:

• “I look forward to things.”

• “I try to stay involved with things”

It was noticed that 3 out of 11 patient participants gave more than 50% neutral or disagreement to the above statements.

**Conclusion:**

During the trial, some patients and their caregivers started to ask about LOVOT and wanted to interact with LOVOT as well. Staff in SCH looked forward to spending time with LOVOT after a long day of work and are already anticipating for the next LOVOT visit. Most respondents had positive experience with LOVOT with patients reporting it as an interactive partner and staff respondents reporting positive well‐being and a good form of stress relief from the hectic work day.

